# Trajectories of eating behaviors in a nationally representative cohort of U.S. adolescents during the transition to young adulthood

**DOI:** 10.1186/s12966-015-0298-x

**Published:** 2015-11-04

**Authors:** Leah M. Lipsky, Denise L. Haynie, Danping Liu, Ashok Chaurasia, Benjamin Gee, Kaigang Li, Ronald J. Iannotti, Bruce Simons-Morton

**Affiliations:** Division of Intramural Population Health Research, Eunice Kennedy Shriver National Institute of Child Health and Human Development, 6100 Executive Blvd, North Bethesda, MD 20852 USA; Department of Health and Exercise Science, Colorado State University, B 215E Moby Complex, Fort Collins, CO 80523 USA; The CDM Group, 7500 Old Georgetown Road, North Bethesda, MD 20814 USA

**Keywords:** Eating behaviors, Body mass index, Emerging adults, Prospective research

## Abstract

**Background:**

Diets of U.S. adolescents and adults do not meet recommendations, increasing risk of chronic disease. This study examined trajectories and predictors of eating behaviors in U.S. youth from age 16–20 years, and evaluated longitudinal associations of eating behaviors with weight outcomes.

**Methods:**

Data come from the first four waves (years) of the NEXT Generation Health Study, a nationally representative cohort of U.S. students in 10^th^ grade during the 2009–2010 school year (*n* = 2785). Annual surveys queried frequency of food group intake (times/day of fruit and vegetables, whole grains, sugar-sweetened soda, sweet and salty snacks), and meal practices (days/week of breakfast, family meals, fast food, and television during meals). Body mass index (BMI, kg/m^2^) was calculated from self-reported height and weight. Adjusted generalized estimating equations and linear mixed models with multiple imputation for missing data estimated eating behavior trajectories overall and by baseline weight status (normal weight = 5 ≤ BMI%ile < 85, overweight = 85 ≤ BMI%ile < 95, obese = BMI%ile ≥ 95), accounting for the complex sampling design. Separate GEE models estimated longitudinal associations of food group frequencies with meal practices and of BMI with eating behaviors.

**Results:**

Eating behaviors tracked strongly from wave 1–4 (residual intraclass correlation = 41 % - 51 %). Across all baseline weight categories, frequency of food group intake and meal practices decreased over time, except for fast food, which remained stable. Fruit/vegetable intake frequency was associated positively with family meals (β ± SE = 0.33 ± 0.05) and breakfast (0.18 ± 0.03), and inversely with fast food (−0.31 ± 0.04), while whole grain intake frequency was associated positively with family meals (0.07 ± 0.02), television meals (0.02 ± 0.009) and breakfast (0.04 ± 0.01). Soda and snacks were positively associated with television meals (0.08 ± 0.008 and 0.07 ± 0.009, respectively) and fast food (0.24 ± 0.02 and 0.20 ± 0.03, respectively), while soda was inversely associated with breakfast frequency (−0.05 ± 0.01). Time-varying BMI was unrelated to eating behaviors other than an inverse association with time-varying snacks (−0.33 ± 0.12).

**Conclusions:**

Strong tracking over time supports the importance of early establishment of health-promoting eating behaviors in U.S. adolescents. Findings suggest meal practices may be important intervention targets. Lack of evidence for hypothesized associations of BMI and eating behaviors indicates the need for research confirming these findings using more precise measures of dietary intake.

**Electronic supplementary material:**

The online version of this article (doi:10.1186/s12966-015-0298-x) contains supplementary material, which is available to authorized users.

## Background

U.S. adolescents consume less than recommended amounts of fruit, vegetables and whole grains, and exceed recommended intake of empty calories [1]. The transition from adolescence to young adulthood is an understudied developmental period characterized by increased independence of behavioral decision-making and the establishment of life-long health behaviors [2]. Given the concomitant increase in obesity [3] and its associated adverse health outcomes [4, 5], research is needed elucidating the variability and determinants of weight-related behaviors during this transition.

Prospective studies evaluating eating behaviors in emerging adults have been conducted primarily in international cohorts and geographically-limited U.S. studies. Findings suggest adolescent dietary behaviors such as intake of fruit and vegetables, sugary foods, and snacks persist somewhat into adulthood [6–9], although studies have reported an overall decrement in diet quality during this period [6, 10]. Evidence indicates that dietary quality is influenced by meal practices such as eating breakfast [11], family meals [12] and fast food [13], and that both dietary quality and meal practices are linked with sociodemographic characteristics [14, 15]. However, prospective research using current, nationally representative samples is needed to evaluate the relevance of these factors in contemporary U.S. adolescents. Furthermore, while excess weight is hypothesized to result from greater frequency of eating behaviors contributing to greater total energy intake [16], few consistent associations have emerged. Additionally, previous studies have not examined whether weight status, which may culminate from previously-established eating behaviors or precipitate modifications of eating behavior for the purpose of weight control, predicts differential eating behavior trajectories. An important limitation of previous studies is the absence of repeated assessments of body weight and eating behaviors in individuals over time, which would enable examining a range of time-specific weight measures for evaluating these pathways, including concurrent weight as well as weight change that precedes a behavior (retrospective), or that follows a behavior (prospective).

Objectives of this study were to examine trends and changes in eating behaviors during the adolescent-adult transition in a contemporary, nationally representative U.S. cohort, and to examine whether these trends differ by sociodemographic factors or baseline weight status. We further sought to examine longitudinal associations among eating behaviors, and of eating behaviors with concurrent body mass index (BMI, kg/m^2^), retrospective and prospective BMI change in order to elucidate the dynamic relationship between eating behaviors and weight outcomes.

## Methods

### Study design and setting

Data come from the first four waves (years) of the NEXT Generation Health Study, an on-going seven-year observational cohort study of multiple health indicators and behaviors in a nationally representative sample of U.S. adolescents followed prospectively. School districts were the primary sampling units, stratified by the nine major U.S. census divisions. Out of 137 schools with 10^th^ grade selected, 81 (59 %) agreed to participate. Classrooms within schools were randomly selected for inclusion. Baseline data were collected during the 2009–2010 school year; however, due to timing of school approval for participation, baseline assessments for 260 of the participants occurred during the second wave (2010–2011, 11^th^ grade). Participants completed self-administered surveys annually. Over the first four waves, survey data were collected from 2785 participants, with 78 % retention in wave 4. Schools with large percentages of African American students were oversampled to obtain reliable estimates for this subgroup; a sufficient number of Hispanic students were obtained to provide reliable subgroup estimates without oversampling. Parents provided informed consent for their child’s participation; youth provided assent (if <18 years of age) or consent (if ≥18 years). Procedures were approved by the institutional review board at the *Eunice Kennedy Shriver* National Institute of Child Health and Human Development.

### Measures

#### Eating behaviors

Eating behavior questions were modified from the Youth Risk Behavior Surveillance System [17] and the multi-national Health Behaviour in School-Aged Children study [18]. Participants were asked “During the past 7 days, how many times did you eat or drink…?” Responses, interpreted as food group intake frequency [19], ranged from never to 4 or more times per day. Food groups included whole grains (assessed waves 2–4), sugar-sweetened soda/pop (soda), sweet and salty snacks (snacks, assessed waves 2–4) and fruit and vegetables. Intake frequency of fruit and vegetables in wave 1 was calculated by summing responses to fruit, 100 % fruit juice, green salad, carrots, and other vegetables (not including potatoes, green salad or carrots); due to modifications to the screener beginning in wave 2, wave 2–4 fruit and vegetable intake frequency was calculated by summing responses to fruit, 100 % fruit juice, green vegetables, orange vegetables, and beans. Meal practices (days/week) queried included breakfast (“How often do you usually have breakfast (more than a glass of milk or fruit juice))”, family meals (“How often do you have an evening meal together with your mother/stepmother or father/stepfather”, assessed waves 1–3), TV meals (“How often do you watch television during a meal at home”, assessed waves 1–3), and fast food (“How often do you eat in a fast food restaurant”). Responses ranged from never to every day.

#### Body weight variables

Body mass index (BMI, kg/m^2^) was calculated at each wave from self-reported height and weight; sex- and age-specific Centers for Disease Control BMI percentiles (%ile) [20] were used to define baseline weight status (underweight = BMI%ile < 5, normal weight = 5 ≤ BMI%ile < 85, overweight = 85 ≤ BMI%ile < 95, obese = BMI%ile ≥ 95) [21]. Prospective 1-year BMI change (next year BMI – current BMI) was calculated for waves 1 through 3; similarly, retrospective 1-year BMI change (current BMI – previous year BMI) was calculated for waves 2 through 4.

#### Covariates

Covariates were selected *a priori* based on previous research on eating behaviors and body weight. Participants reported sex and race/ethnicity at baseline. The previously-validated Family Affluence Scale was calculated based on participant survey responses regarding household car and computer ownership, family vacations, and bedroom sharing [22], and ranges from 0 (low affluence) to 7 (high affluence). Parent-reported educational attainment, ascertained during the consent process, was categorized as less than high school graduate/high school graduate/some college/bachelor’s degree/graduate degree. Baseline school urbanicity was assigned and categorized as urban/suburban/rural. Self-reported previous-week vigorous physical activity (hours per week), assessed at each wave, was included as a covariate given its associations with dietary intake and body weight [23]. Participants were asked “How many hours a week do you usually engage in vigorous physical activity so much that you get out of breath or sweat”, with responses ranging from none to 7 h or more.

### Statistical analysis

Multiple imputation by chained equations, assuming missing-at-random [24, 25] was used for missing variables. The algorithm iteratively imputes missing variables by estimating its distribution conditional on other variables. Fifty imputed datasets were generated using the R package “mice”. Each dataset was analyzed separately and the results were combined using Rubin’s rule in StataSE version 12 (College Station, TX).

Baseline sociodemographics and physical activity were summarized overall and by baseline weight status. Behavioral variables at each wave were summarized, and *p*-values for the unadjusted time trends were calculated using unadjusted linear generalized estimating equations (GEE).

Linear GEE models were used to evaluate the primary research questions. This analytic method enables examining time-varying (variables that change over time, such as eating behaviors) dependent variables in relation to both time-varying and time-constant (variables that do not change over time, such as sex) independent variables, while accounting for the correlation of repeated measures within subjects. Separate models estimated trends in food group intake frequency (fruit and vegetables, soda, whole grains and snacks) and meal practices (family meals, TV meals, breakfast, and eating at a fast food restaurant). Primary predictors included time (wave) and baseline weight status; covariates included baseline sociodemographics and time-varying height and vigorous physical activity. Differential trends by baseline weight status, race/ethnicity and sex were tested in separate models including a multiplicative interaction term of time with the hypothesized modifying variable (e.g., wave*baseline weight status). Tracking was measured by calculating the residual intraclass correlation (between-cluster variation divided by total variation), using linear mixed models with the same covariates as in the main trends analyses [26].

Associations of each time-varying food group intake frequency (dependent variable) with each time-varying meal practice (independent variable) were evaluated using linear GEE models adjusted for wave, time-varying BMI, height, and vigorous physical activity (hours/day), and baseline sociodemographic variables. Finally, associations of weight outcomes (time-varying BMI, prospective 1-y BMI change, and retrospective 1-y BMI change) with time-varying food group intake frequencies and meal practices were investigated using linear GEE models adjusted for wave, time-varying height and vigorous physical activity, and baseline sociodemographic characteristics. All models adjusted for the complex sampling design.

## Results and discussion

Forty percent of participants were overweight (18.9 %) or obese (20.3 %) at baseline (Table 1). Approximately half of participants were non-Hispanic white, and parents of approximately two-thirds of students reported at least some college education. On average, participants reported approximately 3 h/week of vigorous physical activity at baseline.

**Table 1:** Baseline sample characteristics overall and by baseline weight status^a,b^ (*N* = 2785)

			Body Mass Index Percentile (BMI%ile)			
		Overall	<5	≥5, <85	≥85, <95	≥95
			1.51 % (0.004)	59.27 % (0.02)	18.87 % (0.01)	20.35 % (0.01)
Age (years)		16.27 (0.03)	16.18 (0.19)	16.27 (0.03)	16.26 (0.04)	16.27 (0.05)
Family Affluence Scale^c^		5.41 (0.11)	5.75 (0.41)	5.48 (0.11)	5.32 (0.12)	5.22 (0.13)
Race/ethnicity (%)						
	Non-Hispanic White	55.70 (5.92)	64.37 (14.60)	58.12 (6.23)	56.61 (6.51)	47.16 (6.26)
	Non-Hispanic Black	20.29 (4.43)	16.60 (9.76)	19.27 (4.45)	17.73 (5.15)	25.41 (4.57)
	Hispanic	19.29 (3.71)	17.55 (9.63)	17.57 (3.86)	19.50 (5.70)	24.25 (4.15)
	Other	4.82 (1.00)	1.48 (1.33)	5.05 (0.77)	6.16 (3.10)	3.18 (1.10)
Sex (%)						
	Male	45.48 (1.63)	80.34 (8.42)	43.30 (1.65)	43.86 (4.93)	50.77 (3.11)
	Female	54.52 (1.63)	19.66 (8.42)	56.70 (1.65)	56.14 (4.93)	49.23 (3.11)
Parent education (%)						
	< High School	8.68 (2.06)	15.12 (7.88)	7.91 (1.94)	7.46 (3.43)	11.61 (2.44)
	High school graduate	24.91 (2.96)	12.51 (7.32)	22.71 (2.46)	27.51 (3.02)	29.83 (3.70)
	Some college	39.53 (1.74)	35.80 (15.33)	39.15 (2.29)	38.33 (3.17)	42.02 (2.97)
	Bachelor’s degree	14.58 (1.56)	31.79 (14.66)	17.12 (1.94)	10.27 (1.82)	9.91 (1.82)
	Graduate degree	12.30 (1.87)	4.77 (2.79)	13.12 (2.39)	16.43 (3.38)	6.64 (1.80)
Urbanicity (%)						
	Urban	17.36 (7.65)	25.51 (12.56)	17.58 (7.70)	16.95 (7.66)	16.52 (7.58)
	Suburban	48.81 (9.80)	50.36 (16.28)	46.69 (10.00)	53.86 (9.89)	53.86 (10.19)
	Rural	33.83 (7.41)	24.12 (10.52)	35.73 (8.05)	33.16 (7.29)	29.62 (7.02)
Vigorous physical activity (hours/week)		3.06 (0.11)	2.38 (0.32)	3.15 (0.12)	3.11 (0.16)	2.81 (0.17)

^a^Weight status calculated according to BMI%ile cut-offs: <5 underweight; ≥5, <85 normal weight; ≥85, <95 overweight; ≥95 obese

^b^Values are mean or % (SE)

^c^Ranging from 0 (low affluence) to 7 (high affluence)

### Eating behaviors

Mean intake frequency of fruit and vegetables, whole grains, soda and snacks decreased each wave (Table 2). Similarly, decreases over time were observed in mean frequency of meal practices including TV meals and family meals. In contrast, breakfast frequency changed inconsistently over the four waves; wave 4 frequency was 0.61 days/week lower than in wave 1. Mean fast food frequency was relatively stable throughout waves 1–4, increasing by approximately 0.1 days/week by wave 4.

**Table 2:** Unadjusted mean (SE) body mass index (BMI, kg/m^2^), food group intake (times/day), meal behaviors (days/week), and prospective 1-year body mass index (BMI) change by wave

	Wave				
	1	2	3	4	*P-*trend^a^
Food group intake frequency (times/day)					
Fruit and vegetables	3.95 (0.16)	3.91 (0.12)	3.68 (0.12)	3.41 (0.15)	0.004
Whole grains	--	1.24 (0.05)	1.19 (0.05)	1.00 (0.04)	<0.001
Sugar-sweetened soda	1.10 (0.06)	1.02 (0.05)	0.98 (0.05)	0.82 (0.05)	<0.001
Sweet and salty snacks	--	1.26 (0.04)	1.15 (0.04)	0.89 (0.04)	<0.001
Meal practices (days/week)					
Family meals	2.54 (0.07)	2.27 (0.07)	2.02 (0.07)	--	<0.001
Breakfast	4.45 (0.11)	4.29 (0.11)	4.41 (0.10)	3.84 (0.11)	<0.001
Television during meals	3.34 (0.15)	2.99 (0.14)	2.91 (0.11)	--	<0.001
Fast food	1.05 (0.06)	1.06 (0.06)	1.14 (0.07)	1.15 (0.07)	0.03
BMI (kg/m^2^)	23.74 (0.18)	24.32 (0.21)	24.76 (0.25)	25.27 (0.26)	<0.001
Prospective 1-y BMI change^b^	0.58 (0.11)	0.44 (0.11)	0.51 (0.09)	--	0.66
Vigorous physical activity (hours/week)	3.06 (0.11)	2.78 (0.11)	2.69 (0.10)	2.65 (0.11)	<0.001

^a^ *P* for unadjusted time-trend estimated from unadjusted linear generalized estimating equations

^b^Calculated as following wave BMI – current wave BMI, kg/m^2^

### BMI and vigorous physical activity

Mean BMI increased steadily (0.44-0.58 kg/m^2^) each wave, and was within the overweight category for adults at wave 4. Mean vigorous physical activity decreased by 25 min/week (0.42 h) from wave 1 to 4, with the greatest decline (~17 min/week) occurring between the first two waves.

### Tracking and trends in eating behaviors

Within-person correlation accounted for 41 % - 50 % of the residual variance in time-varying eating behaviors in models estimating associations with baseline weight status (Tables 3 and 4). GEE model estimates indicate that, other than stable fast food, frequencies of food group intakes and meal practices decreased over time. Participants with overweight baseline BMI consumed fewer snacks and less frequent breakfasts and family meals overall as compared with those with normal weight baseline BMI, while participants with obese baseline BMI consumed less frequent soda and snacks. Trends were similar by baseline weight category, as evidenced by no statistically significant interactions of baseline weight category and wave (Additional file 1).

**Table 3:** Estimates (βest ± SE) from linear generalized estimating equations (GEE) evaluating associations of time-varying food group intake frequency (times/day) with baseline weight status^a,b^

	Fruit/vegetables		Whole grains		Soda		Snacks	
Independent variables	βest (SE)	*P*	βest (SE)	*P*	βest (SE)	*P*	βest (SE)	*P*
Wave	−0.16 (0.06)	0.01	−0.12 (0.02)	<0.001	−0.09 (0.02)	<0.001	−0.19 (0.02)	<0.001
Baseline weight status (normal weight = ref)								
Underweight	0.06 (0.75)	0.94	0.06 (0.23)	0.80	0.30 (0.18)	0.11	0.02 (0.17)	0.89
Overweight	−0.14 (0.20)	0.51	−0.03 (0.06)	0.57	−0.10 (0.07)	0.15	−0.22 (0.06)	0.003
Obese	0.22 (0.21)	0.32	0.04 (0.08)	0.66	−0.16 (0.07)	0.04	−0.26 (0.07)	0.001
Baseline height (cm)	−0.004 (0.01)	0.75	−0.0001 (0.003)	0.97	0.0004 (0.004)	0.93	0.006 (0.003)	0.10
Sex (male = ref)	−0.02 (0.23)	0.95	−0.02 (0.06)	0.74	−0.20 (0.08)	0.02	0.0004 (0.08)	>0.99
Race/eth (Non-Hispanic White = ref)								
Non-Hispanic Black	1.02 (0.32)	0.003	0.22 (0.08)	0.02	0.36 (0.09)	0.001	0.41 (0.07)	<0.001
Hispanic	0.64 (0.27)	0.03	0.17 (0.09)	0.09	0.05 (0.13)	0.70	−0.003 (0.10)	0.98
Other	0.21 (0.32)	0.51	0.11 (0.12)	0.40	−0.009 (0.19)	0.96	−0.13 (0.15)	0.41
Family Affluence Scale^c^	−0.03 (0.07)	0.66	−0.01 (0.02)	0.62	−0.01 (0.02)	0.66	−0.03 (0.02)	0.17
Parent education (<High school graduate = ref)								
High school graduate	0.11 (0.26)	0.68	0.12 (0.10)	0.25	0.03 (0.11)	0.80	0.05 (0.12)	0.69
Some college	0.11 (0.33)	0.74	0.15 (0.10)	0.14	−0.06 (0.13)	0.65	0.007 (0.12)	0.96
Bachelor’s degree	0.03 (0.35)	0.92	0.24 (0.13)	0.08	−0.24 (0.12)	0.06	0.01 (0.12)	0.93
Graduate degree	0.36 (0.34)	0.31	0.22 (0.12)	0.07	−0.37 (0.11)	0.002	−0.0002(0.11)	>0.99
Urbanicity (urban = ref)								
Suburban	−0.44 (0.25)	0.09	−0.05 (0.08)	0.53	0.16 (0.09)	0.09	0.10 (0.06)	0.13
Rural	−0.29 (0.25)	0.26	0.09 (0.08)	0.27	0.15 (0.08)	0.06	0.08 (0.06)	0.23
Vigorous physical activity (h/week)	0.16 (0.03)	<0.001	0.04 (0.008)	<0.001	−0.03 (0.01)	0.003	−0.02 (0.01)	0.04
Residual intraclass correlation^d^	0.45		0.41		0.44		0.42	

^a^Weight status calculated according to Body Mass Index percentile cut-offs: <5 underweight; ≥5, <85 normal weight; ≥85, <95 overweight; ≥95 obese

^b^Separate models examining each eating behavior outcome, adjusted for all independent variables

^c^Ranging from 0 (low affluence) to 7 (high affluence)

^d^Estimated from linear mixed model with same covariates as GEE models, calculated by dividing between-cluster variation by total variation

**Table 4:** Estimates (βest ± SE) from linear generalized estimating equations examining associations of time-varying meal practices (days/week) with baseline weight status^a,b^

	Family meal		Breakfast		TV meals^c^		Fast food	
Independent variables	βest (SE)	*P*	βest (SE)	*P*	βest (SE)	*P*	βest (SE)	*P*
Wave	−0.24 (0.03)	<0.001	−0.18 (0.05)	0.001	−0.22 (0.04)	<0.001	0.03 (0.02)	0.05
Baseline weight status (normal weight = reference)								
Underweight	−0.09 (0.36)	0.80	0.10 (0.63)	0.87	−0.56 (0.42)	0.20	0.03 (0.25)	0.91
Overweight	−0.30 (0.12)	0.02	−0.42 (0.11)	0.001	0.10 (0.19)	0.60	0.04 (0.09)	0.69
Obese	−0.28 (0.20)	0.17	−0.24 (0.12)	0.06	0.13 (0.19)	0.51	−0.10 (0.09)	0.26
Baseline height (cm)	−0.008 (0.005)	0.17	0.006 (0.009)	0.48	−0.005 (0.008)	0.53	0.005 (0.004)	0.23
Sex (male = reference)	−0.13 (0.10)	0.18	0.18 (0.14)	0.22	−0.23 (0.15)	0.13	−0.15 (0.05)	0.01
Race/ethnicity (Non-Hispanic White = reference)								
Non-Hispanic Black	−0.33 (0.15)	0.04	−0.30 (0.20)	0.13	1.47 (0.15)	<0.001	0.54 (0.08)	<0.001
Hispanic	−0.16 (0.12)	0.20	0.05 (0.21)	0.80	0.26 (0.19)	0.19	0.13 (0.13)	0.33
Other	0.07 (0.21)	0.72	−0.13 (0.26)	0.63	−0.21 (0.37)	0.58	−0.02 (0.15)	0.88
Family Affluence Score^d^	0.07 (0.04)	0.046	0.01 (0.03)	0.68	−0.02 (0.04)	0.63	0.08 (0.02)	0.002
Parent education (<High school graduate = reference)								
High school graduate	0.07 (0.17)	0.68	0.07 (0.25)	0.78	−0.12 (0.25)	0.64	0.05 (0.13)	0.68
Some college	0.10 (0.18)	0.60	−0.01 (0.28)	0.97	0.12 (0.29)	0.68	0.03 (0.12)	0.83
Bachelor’s degree	0.18 (0.23)	0.44	0.27 (0.29)	0.36	−0.26 (0.22)	0.25	−0.08 (0.11)	0.49
Graduate degree	0.29 (0.18)	0.13	0.50 (0.25)	0.07	−0.74 (0.21)	0.003	−0.17 (0.11)	0.16
Urbanicity (urban = reference)								
Suburban	0.01 (0.15)	0.93	−0.03 (0.18)	0.88	0.03 (0.17)	0.86	0.02 (0.09)	0.81
Rural	−0.05 (0.13)	0.70	0.07 (0.18)	0.71	0.01 (0.16)	0.95	−0.13 (0.11)	0.26
Vigorous physical activity (hours/week)	0.07 (0.02)	<0.001	0.13 (0.02)	<0.001	−0.05 (0.02)	0.03	−0.02 (0.01)	0.15
Residual intraclass correlation^e^	0.51		0.50		0.45		0.48	

^a^Weight status calculated according to Body Mass Index percentile cut-offs: <5 underweight; ≥5, <85 normal weight; ≥85, <95 overweight; ≥95 obese

^b^Separate models examining each eating behavior outcome, adjusted for all independent variables

^c^Watching television during meals

^d^Ranging from 0 (low affluence) to 7 (high affluence)

^e^Estimated from linear mixed models with same covariates as GEE models, calculated by dividing between-cluster variation by total variation

#### Covariates

GEE models indicate few consistent relationships between time-varying eating behaviors and baseline sociodemographic variables (Tables 3 and 4). Non-Hispanic black participants reported higher intake frequency of all food groups, greater frequency of TV meals and fast food, and less frequent family meals versus non-Hispanic white participants. Greater family affluence was associated with more frequent family meals and fast food. Time-varying vigorous physical activity was associated with more frequent fruit and vegetables and whole grains, less frequent soda and snacks, more frequent family meals and breakfast, and less frequent TV meals (Tables 3 and 4). The association of physical activity with fast food was not statistically significant.

Eating behavior trends were mostly non-differential by baseline race/ethnicity and sex, except that breakfast frequency increased over time for females relative to males, and fast food frequency decreased over time for non-Hispanic black relative to non-Hispanic white participants (Additional file 2).

### Associations among eating behaviors

In GEE models examining associations of time-varying food group intake frequency with time-varying meal practices, family meals and breakfast were positively associated with fruit/vegetables and whole grains (Table 5). More frequent breakfast was additionally associated with less frequent soda intake, while more frequent fast food was associated with less frequent fruit/vegetables and more frequent soda and snacks. More frequent TV meals was associated with more frequent soda, whole grains and snacks.

**Table 5:** Estimates from linear generalized estimating equations examining associations of time-varying food group intake frequencies (times/day) with time-varying meal practices (days/week)^a^

	Food group intake frequency (times/day)							
	Fruit/vegetables		Soda		Whole grains		Snacks	
Independent variables	βest (SE)	*P*	βest (SE)	*P*	βest (SE)	*P*	βest (SE)	*P*
Meal practice (days/week)								
Family meals	0.33 (0.05)	<0.001	−0.003 (0.02)	0.89	0.07 (0.02)	<0.001	0.005 (0.02)	0.80
Breakfast	0.18 (0.03)	<0.001	−0.05 (0.01)	<0.001	0.04 (0.01)	0.001	−0.01 (0.009)	0.14
TV meals^b^	0.001 (0.03)	0.96	0.08 (0.008)	<0.001	0.02 (0.009)	0.02	0.07 (0.009)	<0.001
Fast food	−0.31 (0.04)	<.001	0.24 (0.02)	<0.001	−0.03 (0.02)	0.10	0.20 (0.03)	<0.001

^a^Estimates from separate models predicting time-varying intake frequency of fruit/vegetables, soda, whole grains, and sweet/salty snacks (times/day). Models adjusted for wave, time-varying body mass index, height, and vigorous physical activity (hours/day), and baseline sex, race/ethnicity, family affluence score (ranging from 0 = low affluence to 7 = high affluence), parent education, and urbanicity

^b^Watching television during meals

#### Associations of BMI with eating behaviors

Time-varying BMI was unrelated to time-varying eating behaviors, other than an inverse relationship with snacks (Tables 6 and 7). Time-varying BMI of non-Hispanic black and Hispanic participants was between 1.97-2.69 kg/m^2^ and 1.27-1.54 kg/m^2^ higher, respectively, than that of non-Hispanic white participants, and was not associated with other sociodemographic variables. BMI was inversely associated with vigorous physical activity; each additional hour of vigorous physical activity was associated with an approximately 0.1 kg/m^2^ lower BMI. Eating behaviors were not associated with prospective or retrospective 1-year BMI change (Additional file 3).

**Table 6:** Estimates from linear generalized estimating equation models examining associations of time-varying body mass index (BMI, kg/m^2^) with time-varying food group intake frequencies (times/day)^a^

	Model 1		Model 2		Model 3		Model 4	
Independent variables	βest (SE)	*P*	βest (SE)	*P*	βest (SE)	*P*	βest (SE)	*P*
Fruit and vegetables (times/day)	0.09 (0.06)	0.16	--		--		--	
Whole grains (times/day)	--		0.14 (0.15)	0.35	--		--	
Soda (times/day)	--		--		-0.15 (0.12)	0.23	--	
Snacks (times/day)	--		--		--		-0.33 (0.12)	0.02
Wave (year)	0.49 (0.05)	<0.001	0.47 (0.05)	<0.001	0.48 (0.06)	<0.001	0.40 (0.07)	<0.001
Height (cm)	0.02 (0.02)	0.47	0.01 (0.02)	0.48	0.02 (0.02)	0.52	0.02 (0.02)	0.48
Sex (male=ref)	0.11 (0.52)	0.84	0.07 (0.54)	0.89	0.12 (0.58)	0.83	0.12 (0.58)	0.84
Race/ethnicity (Non-Hispanic White=ref)								
Non=Hispanic Black	2.22 (0.82)	0.01	2.36 (0.86)	0.01	2.53 (0.94)	0.01	2.69 (0.95)	0.009
Hispanic	1.36 (0.57)	0.03	1.43 (0.59)	0.02	1.52 (0.60)	0.02	1.54 (0.61)	0.02
Other	0.46 (0.69)	0.51	0.48 (0.69)	0.49	0.87 (0.80)	0.29	0.84 (0.80)	0.30
Family Affluence Score^b^	-0.22 (0.23)	0.33	-0.23 (0.23)	0.33	-0.24 (0.27)	0.37	-0.25 (0.26)	0.35
Parent education (<High school graduate=ref)								
High school graduate	0.26 (0.66)	0.70	0.27 (0.65)	0.68	0.38 (0.68)	0.58	0.41 (0.68)	0.55
Some college	0.59 (0.65)	0.37	0.59 (0.65)	0.38	0.78 (0.71)	0.29	0.80 (0.70)	0.27
Bachelor’s degree	-0.75 (0.83)	0.37	-0.78 (0.84)	0.36	-0.70 (0.88)	0.43	-0.65 (0.87)	0.46
Graduate degree	0.05 (0.96)	0.96	0.03 (0.99)	0.98	0.24 (1.02)	0.82	0.27 (1.02)	0.79
Urbanicity (urban=ref)								
Suburban	1.27 (0.70)	0.08	1.26 (0.69)	0.08	1.46 (0.76)	0.07	1.48 (0.76)	0.06
Rural	1.07 (0.58)	0.08	1.07 (0.57)	0.07	1.24 (0.64)	0.06	1.28 (0.64)	0.06
Vigorous physical activity (hours/week)	-0.13 (0.04)	0.006	-0.12 (0.04)	0.008	-0.15 (0.05)	0.006	-0.15 (0.05)	0.006

^a^Each model examined associations of time-varying BMI outcome with a single food group, adjusted for wave, time-varying height and vigorous physical activity, and baseline sociodemographic characteristics

^b^Ranging from 0 (low affluence) to 7 (high affluence)

**Table 7:** Estimates from linear generalized estimating equation models examining associations of time-varying body mass index (BMI, kg/m^2^) with time-varying meal practices (days/week)^a^

	Model 5		Model 6		Model 7		Model 8	
Independent variables	βest (SE)	*P*	βest (SE)	*P*	βest (SE)	*P*	βest (SE)	*P*
Family meals	−0.12 (0.10)	0.24	--		--		--	
Breakfast	--		−0.05 (0.05)	0.25	--		--	
Fast food restaurant	--		--		−0.10 (0.11)	0.36	--	
TV meals^b^	--		--		--		0.08 (0.05)	0.11
Wave (year)	0.45 (0.06)	<0.001	0.48 (0.05)	<0.001	0.49 (0.06)	<0.001	0.47 (0.05)	<0.001
Baseline height (cm)	0.01 (0.02)	0.58	0.02 (0.02)	0.47	0.01 (0.02)	0.54	0.02 (0.02)	0.47
Sex (male = ref)	0.01 (0.48)	0.98	0.09 (0.52)	0.87	0.05 (0.48)	0.92	0.11 (0.53)	0.83
Race/ethnicity (Non-Hispanic White = ref)								
Non-Hispanic Black	2.04 (0.84)	0.02	2.36 (0.87)	0.01	1.97 (0.81)	0.02	2.30 (0.87)	0.02
Hispanic	1.27 (0.57)	0.04	1.43 (0.58)	0.02	1.27 (0.57)	0.04	1.42 (0.58)	0.02
Other	0.29 (0.65)	0.67	0.48 (0.68)	0.49	0.29 (0.66)	0.66	0.47 (0.68)	0.49
Family Affluence Score^c^	−0.20 (0.20)	0.32	−0.22 (0.23)	0.35	−0.21 (0.20)	0.31	−0.22 (0.23)	0.34
Parent education (<High school graduate = ref)								
High school graduate	0.20 (0.65)	0.76	0.27 (0.65)	0.68	0.20 (0.65)	0.76	0.27 (0.65)	0.68
Some college	0.48 (0.65)	0.46	0.60 (0.65)	0.37	0.46 (0.65)	0.48	0.60 (0.66)	0.37
Bachelor’s degree	−0.79 (0.79)	0.33	−0.75 (0.82)	0.37	−0.80 (0.80)	0.33	−0.73 (0.82)	0.38
Graduate degree	0.02 (0.95)	0.99	0.07 (0.97)	0.95	0.004 (0.97)	>0.99	0.11 (0.96)	0.91
Urbanicity (urban = ref)								
Suburban	1.10 (0.65)	0.10	1.23 (0.69)	0.08	1.10 (0.65)	0.10	1.23 (0.69)	0.09
Rural	0.93 (0.54)	0.10	1.03 (0.57)	0.08	0.93 (0.54)	0.10	1.05 (0.57)	0.08
Vigorous physical activity (hours/week)	−0.11 (0.03)	0.002	−0.12 (0.04)	0.01	−0.12 (0.04)	0.003	−0.11 (0.04)	0.01

^a^Separate models examined associations of time-varying BMI outcome with each meal practice, adjusted for wave, time-varying height and vigorous physical activity, and baseline sociodemographic characteristics

^b^Watching television during meals

^c^Ranging from 0 (low affluence) to 7 (high affluence)

## Conclusions

In this nationally representative cohort of U.S. adolescents, eating behaviors tracked strongly between 16–20 years of age, although small decreases were observed over this period in intake frequency of food groups including fruit and vegetables, whole grains, sweet and salty snacks (snacks), sugar-sweetened carbonated beverages (soda), and of meal practices including family meals, watching television during meals (TV meals), and breakfast. In contrast, frequency of eating at a fast food restaurant (fast food) remained constant over follow-up. Trends were similar across baseline weight status, both sexes, and all races/ethnicities. There was little evidence of associations of these eating behaviors with weight outcomes.

This study provides unique findings regarding the consistency of eating behaviors in emerging adults in the United States assessed annually during the transition from adolescence to adulthood. Estimates of residual intraclass correlation coefficients suggest time-invariant subject-specific characteristics account for approximately 50 % of the variance of time-varying eating behaviors not explained by covariates [26]. Although estimates are somewhat higher than previous reports, comparisons are difficult given the differences in the assessed eating behaviors and the methods used to estimate tracking [9, 27]. In the ASH30 study of 198 participants in North East England, unadjusted Pearson correlation coefficients of baseline (mean age 11.6 years) and follow-up (mean age 32.5) intakes of fruit, vegetables, bread and starches ranged from r = 0.22 – 0.26 [9]. Similarly lower adjusted tracking coefficients were obtained by estimating associations of time-varying eating behaviors with baseline values for intakes of fruit (β = 0.33, 95 % CI = 0.25, 0.41) and vegetables (β = 0.27, 95 % CI = 0.19, 0.36) in the Amsterdam Growth and Health Longitudinal Study, which followed participants from age 13 to 36 years [27]. In addition to differences in statistical methods for estimating tracking, the longer duration of follow-up (approximately 20 years), the younger and more developmentally distinct baseline age (11–13 years), and different environmental settings of previous studies likely contribute to discrepancies with the current findings. The present study reports the first tracking estimates in emerging adults for meal practices including frequency of breakfast, family meals, TV meals and fast food, which were similar to those of food group intake frequencies. Taken together, the large amount of within-person correlation observed across a range of time-varying eating behaviors suggests the importance of early establishment of health-promoting eating behaviors.

Few prospective studies of U.S. adolescents have reported associations of eating behaviors with body weight in emerging adults. Despite modestly lower overall frequencies of snacks, breakfast and family meals in participants with baseline overweight vs. normal weight, and of snacks and soda in obese participants, eating behavior trends during the transition to young adulthood did not vary by baseline weight status. Additionally, neither prospective nor retrospective 1-year BMI change was associated with any assessed eating behavior, indicating that BMI did not predict progression of eating behaviors, and that these eating behaviors did not predict BMI change. Results based on a similar eating behavior assessment from the multi-national Health Behaviour in School-Aged Children study indicated no cross-sectional associations of these eating behaviors with odds of being overweight in 11-, 13- and 15-year-olds other than an inverse association with breakfast frequency among males [28]. Other than an overall inverse association in the current study of time-varying BMI with time-varying snack frequency, eating behaviors were not related to time-varying BMI in longitudinal models, consistent with previously-reported null associations [8, 29–31]. The modest inverse association of BMI and snacks was opposite to the hypothesized direction, given these snacks are energy dense foods [32] that would be hypothesized to contribute to positive energy balance [33]. Observational studies have produced mixed findings regarding associations of snacking with weight outcomes [34, 35], while limited experimental evidence suggests that altering snacking frequency has no noticeable impact on body weight [36]. It is unlikely that lower reported snack intake occurred as a behavioral response to high BMI, given the null association of snack intake with retrospective BMI change. Findings are also inconsistent with differential social desirability bias or underreporting by BMI, given that similar associations were not consistently observed for any other eating behavior. One consideration is whether the available metric for snacking (times/day) was sufficient to capture energy intake from these foods, which would impact the ability to detect associations with weight outcomes. Additional studies are needed to replicate this finding and explore mechanisms.

Despite the observed consistency of eating behaviors within participants, eating behaviors decreased modestly between late adolescence and young adulthood other than stable fast food frequency. The findings are somewhat dissimilar from others [6, 10], which have indicated an overall decrement in diet quality in the transition to young adulthood. The decline in intake frequency of most food groups may reflect an overall decrease in food intake; however, this cannot be confirmed from these data. Trends observed in the present study do not suggest increased social desirability bias over time when considering that trends were similar for both health-promoting and non-health-promoting eating behaviors as well as for vigorous physical activity. Additionally, the similar eating behavior trends observed by race/ethnicity and sex do not replicate previous findings [15, 37], and the more frequent intake of all food groups in non-Hispanic black versus non-Hispanic white participants does not reflect an overall discrepancy in diet quality, as previously reported [14, 15]. Vigorous physical activity, included as a covariate given its relationship with dietary intake and energy balance [23], was associated with more optimal eating behaviors, including more frequent fruit and vegetables and whole grains, less frequent soda and snacks, more frequent breakfast and family meals, and fewer TV meals. Physical activity was similarly associated with lower soda and sugar-sweetened beverage intake in an international youth cohort [8]; however, the current findings represent the first reported associations of physical activity with a range of eating behaviors based on prospective data from a nationally representative sample of U.S. adolescents. Although the magnitude of these associations appears small, the consistency of the association of vigorous physical activity with several more health-promoting eating behaviors warrants further investigation.

Findings indicate associations of greater frequency of family meals and breakfast with more optimal food group intake frequency (e.g., more fruit and vegetables and whole grains, less soda), and of increased fast food and TV meals with less optimal food group intake frequency (e.g., less fruit and vegetables, more soda and snacks). Similar associations of fruit and vegetable intake with fast food intake, time watching television, and family meal frequency were observed in the Project EAT study, although these associations were not statistically significant once models adjusted for baseline intake [38]. Similar associations have been observed of diet quality with TV meals [39, 40], breakfast [41, 42], fast food [43, 44] and family meals [12, 37] . These observations support findings from qualitative research indicating the importance of context-specific environmental, social and situational influences on food choices [45], which are manifested by differences in dietary intake [46]. Experimental trials are needed to determine how modifying the food choices or frequency of specific eating contexts could lead to improvements in overall diet quality.

Several limitations should be considered when interpreting these findings. First, the self-report screener assessing eating behaviors is more susceptible to measurement error than alternative assessment methods [47]. Recent estimates of daily fruit and vegetable intake from a screener used as the basis for the version used in the current study were 0.5-1.3 times/day greater than those obtained from the estimated servings/day from 24-h diet recalls, although Pearson correlations ranged from *r* = 0.14-0.26 [19]. However, the screener is considered adequate for population-level surveillance of eating behaviors, and in the context of a large, prospective study evaluating a variety of health behaviors of public health significance, a short diet screener was required due to its low cost and respondent burden [19]. Self-report assessment for other variables is also susceptible to recall bias, social desirability bias, and errors of self-estimation. Though BMI was based on self-reported height and weight, it correlates strongly (*r* = 0.92) with measured BMI in adolescents [48], and is strongly associated with body fat [49] and cardiovascular risk factors [50] in this population. In addition, while we adjusted for self-reported vigorous physical activity, a precise measure of total energy expenditure was not available. Further, the observational design precludes inferences regarding causation. Despite these limitations, this study has notable strengths, including a large, prospective, nationally representative sample assessed annually for four years, and sufficient sample size to obtain generalizable estimates for non-Hispanic white, non-Hispanic black and Hispanic American youth. The nearly 80 % retention rate at wave 4, the use of multiple imputation for missing data, and the ability to adjust for several hypothesized covariates support the internal validity of the findings. Finally, the prospective design enables examination of the temporality of associations, an important advantage over cross-sectional designs.

The strong tracking of a range of eating behaviors from late adolescence to early adulthood observed in this nationally-representative sample suggests the potential for effective dietary interventions to have sustained impact during this critical developmental window. Other than stable fast food frequency, participants reported modest decreases in a range of health-promoting and non-health-promoting eating behaviors, suggesting the need for additional research elucidating the intake patterns of emerging adults. Similar eating behavior trends regardless of baseline weight status and sociodemographic characteristics underscore the importance of supporting optimal eating behaviors in all subgroups. We found no support for hypothesized associations of these eating behaviors with weight outcomes, indicating the need to investigate additional behavioral correlates in future research of weight outcomes in emerging adults. However, associations of meal practices (greater frequency of breakfast, family meals, and less frequent TV meals and fast food) with greater frequency of health-promoting food group intake suggest potential target behaviors for intervention development. Additional studies are warranted using more precise measures of dietary intake to investigate to what extent these behaviors reflect overall diet quality.

## Additional files

Additional file 1:
**“Estimates (β ± SE) from linear generalized estimating equation models examining associations of eating behavior trends over time by baseline weight status”, contains results from models examining eating behavior trends by baseline weight status.** (XLSX 10 kb) Additional file 2:
**“Estimates (β ± SE) from linear generalized estimating equation models examining associations of eating behavior trends over time by sex and race/ethnicity,” contains results from models examining eating behavior trends by sex and race/ethnicity.** (XLSX 13 kb) Additional file 3:
**“Estimates (β ± SE) from linear generalized estimating equations predicting retrospective and prospective** **1-year** **body mass index (BMI, kg/m** ^2^ **) change,” contains results from models examining longitudinal associations of eating behaviors with change in BMI.** (XLSX 12 kb)

## Abbreviations

BMI: Body mass index
Fast food: eating at a fast food restaurant
GEE: generalized estimating equations
Snacks: sweet and salty snacks
Soda: sugar-sweetened soda/pop
TV meals: watching television during meals
